# Recent Advances and Environmental Challenges in Polymer Nanocomposites: Nanofillers, Processing Technologies, and Applications

**DOI:** 10.3390/polym18172120

**Published:** 2026-08-31

**Authors:** Dinghao Wang, Olena Bakulich, Viacheslav Trachevskyi, Mingyang Ta, Andrii Bieliatynskyi

**Affiliations:** 1 School of Civil Engineering, North Minzu University, 204 Wenchang Road, Yinchuan 750021, China; 20247582@stu.nmu.edu.cn (D.W.); tmy_1258@126.com (M.T.); beljatynskji@ukr.net (A.B.); 2 Faculty of Management, Logistics and Tourism, National Transport University, 1 Mykhailo Omelianovych-Pavlenko Str., 01010 Kyiv, Ukraine; 3 Institute of Macromolecular Chemistry of the National Academy of Sciences of Ukraine, 48 Kharkivske Hwy, 02155 Kyiv, Ukraine; meches49@ukr.net

**Keywords:** ecological safety, interphase interaction, nanofillers, nanotechnologies, polymer nanocomposites

## Abstract

Polymer nanocomposites have attracted considerable attention owing to their ability to achieve substantial improvements in mechanical, thermal, electrical, barrier, and multifunctional properties through the incorporation of low concentrations of nanoscale fillers. This review provides a comprehensive analysis of recent advances in polymer nanocomposites, focusing on the relationships between nanofiller characteristics, processing strategies, interfacial interactions, and the resulting material performance. Different classes of nanofillers, including carbon-based, ceramic, metallic, polymeric, and hybrid nanostructures, are systematically compared with respect to their morphology, surface chemistry, of processing routes, including melt blending, solution processing, in situ polymerization, and surface functionalization, on nanoparticle dispersion and polymer–nanofiller interfacial adhesion is critically discussed. The review further evaluates how these factors govern the mechanical, thermal, electrical, dielectric, and barrier properties of polymer nanocomposites and summarizes their applications in aerospace, automotive engineering, electronics, biomedical devices, energy systems, construction, and advanced packaging. Current technological challenges, including nanoparticle aggregation, long-term stability, process scalability, environmental impact, and nanomaterial safety, are also examined. Finally, emerging research directions, including hybrid nanofillers, sustainable polymer systems, digital materials design, and machine-learning-assisted optimization of polymer nanocomposites, are highlighted. This review provides an integrated perspective on the design and processing of high-performance polymer nanocomposites and identifies key opportunities for future research and industrial implementation.

## 1. Introduction

In the 21st century, high-tech materials have become crucial due to the demands of industry, energy, medicine, and sustainable development initiatives. Society is seeking to minimize environmental impact while maximizing the energy efficiency, durability, and functionality of materials [1]. Polymeric composite materials modified with nanofillers are emerging as a key component of a new generation of structural, functional, and intelligent materials [2]. Developing such materials aims to increase the strength-to-mass ratio, improve chemical and thermal resistance, enhance electrical conductivity, promote biocompatibility, and equip materials with self-organization capabilities. This opens up new possibilities for their application in various industries.

Typically, microdispersed fillers, such as glass and carbon fibers, talc, kaolin, quartz, graphite, and metal powders, have been widely used to improve the mechanical, thermal, and performance characteristics of polymers. Such materials allow for a significant increase in the stiffness, strength, and heat resistance of polymer composites; however, achieving the required level of properties usually requires the addition of a substantial amount of filler (15–40 wt.% or more). An increase in the concentration of microparticles is accompanied by an increase in material density, a deterioration in melt flowability, more difficult processing, and an increased likelihood of localized stress concentrations, which can lead to crack initiation and premature failure of the products.

Furthermore, the effectiveness of traditional microdispersed fillers is greatly limited by their relatively small specific surface area, which reduces the contact area between the filler and the polymer matrix and limits the ability to form a strong interphase layer. In most cases, such composites improve only certain mechanical properties, while simultaneously enhancing the material’s electrical conductivity, thermal conductivity, barrier properties, or functional activity is quite difficult to achieve. It is precisely these limitations that have been the main reason for the active shift toward the use of nanoscale fillers, which are capable of providing comprehensive improvements in properties at significantly lower concentrations.

Unlike traditional microdispersed fillers, which mainly exhibit mechanical reinforcement [3,4], nanofillers with particle sizes less than 100 nm have a high specific surface area and unique physical and chemical properties that manifest at low volume contents (typically 1–5%) [2,5]. These effects are explained by quantum effects, the specifics of interfacial interaction, and the ability to form nanoscale networks within the polymer. This changes the mechanisms of crack propagation, heat transfer, sorption, and diffusion [2]. Introducing nanoparticles into polymer matrices modifies the mechanical, optical, magnetic, and electrical properties of composites, enabling the creation of materials with specified functional characteristics at the nanoscale [2,5].

Analytical reports confirm the economic feasibility of the polymer materials market. The global polymer market was estimated at approximately USD 705 billion in 2022 and is projected to exceed USD 1.157 trillion by 2032, growing at an average annual rate of about 5.2% from 2023 to 2032 [6]. Recent comprehensive review studies indicate that the current development of polymer nanocomposites is linked not only to the creation of new types of nanofillers but also to the refinement of methods for characterizing them, modeling interfacial interactions, and predicting their performance properties. Particular attention is paid to a comprehensive analysis of the structure, morphology, and mechanical and thermal characteristics of materials using complementary research methods, which makes it possible to establish a relationship between the manufacturing technology, microstructure, and final properties of polymer nanocomposites. This integrated approach is currently regarded as one of the key areas of development in modern polymer materials science [7].

Nanomodified polymers are used in aviation and space technology because lightweight composites reduce the weight of structures without compromising strength. This helps reduce fuel consumption and increase aircraft efficiency [8]. In the automotive industry, there is a growing demand for materials that improve safety and reduce vehicle weight. In medicine, polymeric nanocomposites are used to create biocompatible implants, smart drug delivery systems, and tissue engineering carriers. In electronics, nanotubes, graphene, and other innovative fillers form the basis for flexible conductors, sensors, and nanoelectronic components, providing opportunities for shielding electromagnetic interference [9,10].

However, the industry faces several challenges: achieving a homogeneous distribution of nanofillers in the polymer matrix, ensuring the long-term stability of nanostructures, and preventing the aggregation of nanoparticles, particularly under operational loads [5]. Scaling up production processes to industrial volumes without losing control over morphology and interfacial properties remains urgent. Incomplete knowledge of the mechanisms of property formation in multicomponent nanosystems and the unpredictability of polymer chain interaction with nanoparticles indicate the need for consistent approaches to predicting composite behavior [11]. Furthermore, issues related to health and environmental safety arise. Therefore, the potential toxicity of nanomaterials, their biodegradation, and their impact on the environment and humans throughout the entire service life of products must be assessed in depth [1,5].

An analysis of the literature reveals an imbalance in the focus of research. While most publications are devoted to synthesizing new nanofillers or testing their properties in the laboratory, they often lack systematic, interdisciplinary approaches that integrate knowledge of chemistry, physics, materials science, biotechnology, and nanomechanics [2]. Although new directions have emerged since 2019, including research on biodegradable polymer matrices, green chemistry, and scalable synthesis methods [12,13], the overall imbalance in research focus remains largely unchanged. The vast majority of works continue to focus on mechanical characteristics, while environmental, applied, and long-term reliability aspects are covered in a fragmented manner.

There is an insufficient number of comparative studies demonstrating how the structural and morphological characteristics of different types of nanofillers determine the final properties of composites in real-world applications. Analyzing the effectiveness of different fillers in specific industries and assessing the technological limitations and economic feasibility of implementing them is critical to determining priority application areas and prospects for further development [2]. A key gap is the absence of an interdisciplinary, comparative analysis of nanofiller properties in an applied context that summarizes structural and morphological features and their impact on the final characteristics of composites in real-world applications.

In this regard, the aim of this review article is to provide a comprehensive analysis of the current state of research on polymer nanocomposites (hereinafter—PNCs) in order to establish the relationship between the nature of nanofillers, the characteristics of their interaction with the polymer matrix, manufacturing technologies, and the range of performance properties of the materials. Particular attention is paid to a critical analysis of current approaches to the characterization, modeling, and practical application of polymer nanocomposites, as well as to identifying prospects for their further development.

In contrast to previous reviews [2,14], this paper focuses on an interdisciplinary approach that combines materials science, engineering, environmental, and economic aspects of PNCs. The review features a critical discussion of knowledge gaps, a comparative analysis of key properties, and the use of visualizations (tables, diagrams, and figures) to present the current state of research and prospects for practical application in a more systematic manner. The paper is based on foundational works in nanomaterials science and polymer physics, as well as modern experimental and analytical studies from the last five years, prioritizing English-language sources. It includes diagrams and tables that visualize the structure of nanofillers, their properties, and their range of applications. These visualizations will reveal the interdisciplinary connections and practical applications of PNCs [8,15].

## 2. Materials and Methods

The methodological basis is a systematic, transparent, and reproducible analytical approach involving the sequential implementation of five research stages. Each stage has clearly defined objectives, criteria, and tools that facilitate a thorough analysis of PNCs within an interdisciplinary framework. The first stage involved creating the source base. A literature search was conducted in six authoritative academic databases: Scopus, Web of Science, ScienceDirect, SpringerLink, IEEE Xplore, and Google Scholar. Publications were identified using combinations of keywords related to PNCs, nanofillers, morphology, mechanical properties, electrical conductivity, barrier properties, nanotoxicity, and surface functionalization to increase the relevance. Boolean operators (AND, OR) were used to refine the search strategy and maximize the retrieval of relevant publications. The search primarily included peer-reviewed journal articles published in English. The inclusion criteria were the relevance of the topic; availability of experimental or substantiated numerical data; interdisciplinary approach; the publication in a peer-reviewed journal with an impact factor of at least 1.5 (Q1–Q2); the sufficient citation of the article and its impact in the relevant scientific field. 812 sources were identified at this stage, primarily published between 2015 and 2024.

**Figure 1:**
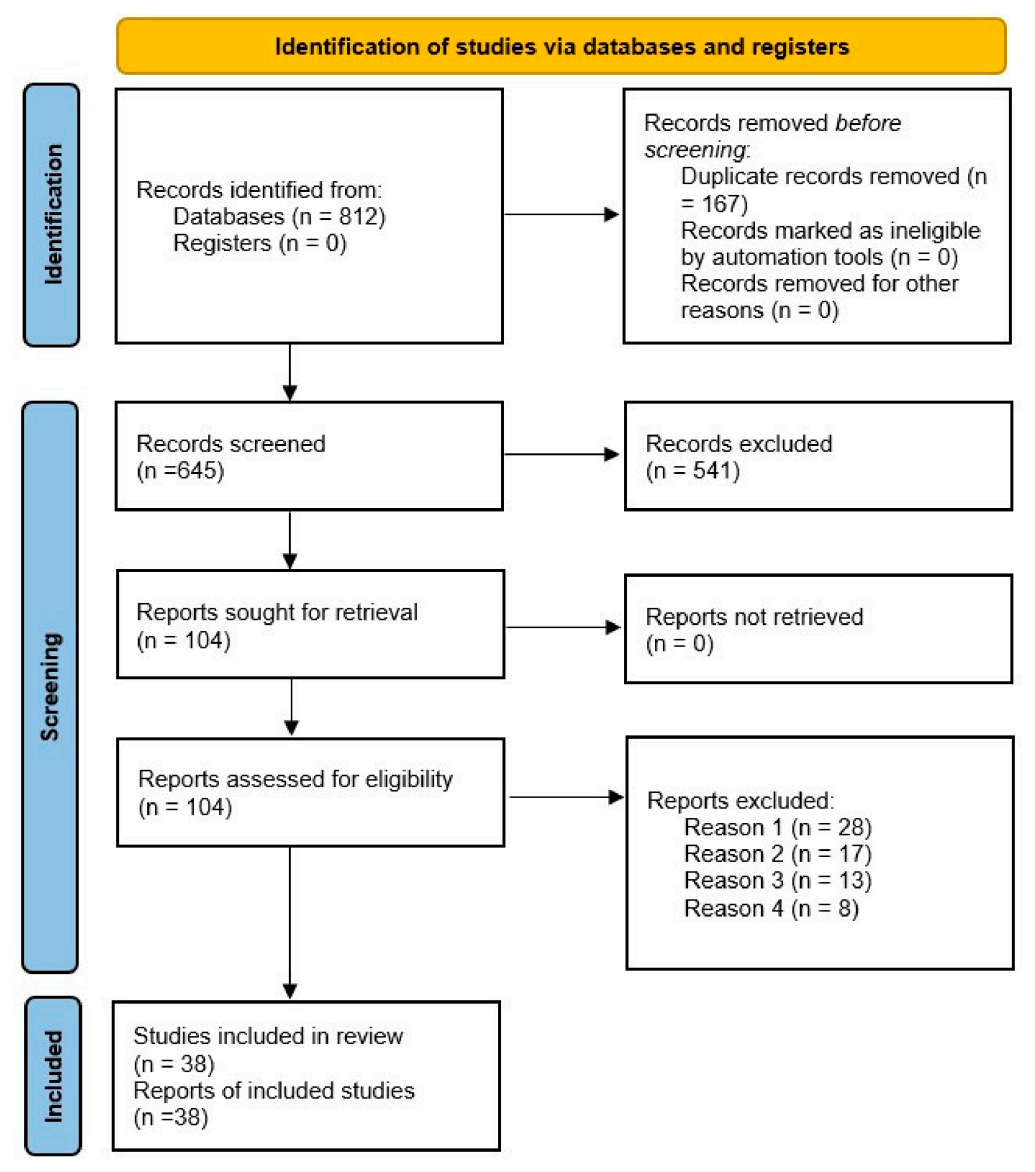
PRISMA 2020 flow diagram illustrating the identification, screening, eligibility assessment, and inclusion of studies in this review.

The identified records were exported from the selected databases and screened according to the Preferred Reporting Items for Systematic Reviews and Meta-Analyses (PRISMA) 2020 Statement [16]. Duplicate records were removed before title and abstract screening. Publications that were clearly unrelated to polymer nanocomposites or did not satisfy the predefined inclusion criteria were excluded. The remaining articles underwent full-text assessment for eligibility. Publications were excluded if they (1) were outside the scope of polymer nanocomposites, (2) consisted of conference papers, editorials, patents, or book chapters without sufficient peer-reviewed scientific content, (3) contained insufficient technical or experimental information, or (4) represented duplicate or substantially overlapping publications. Following the eligibility assessment, 38 studies met the selection criteria and were included in the qualitative synthesis presented in this review. The complete study selection process is summarized in Figure 1 (Table S1).

The second stage was the thematic classification of sources. All selected information was grouped into five thematic clusters that determine the logic of the main part of the article: types of nanofillers (carbon, metal, ceramic, polymer, hybrid); methods of synthesis and integration (precipitation, sol–gel, laser ablation, chemical reduction, extrusion, etc.); properties of nanocomposites (mechanical strength, thermal stability, electrical and thermal conductivity, permeability to gases, optical transparency); applications (aviation, medicine, electronics, energy, packaging); and technological challenges (aggregation, matrix compatibility, biosafety, scaling) [17,18].

The third stage was a comparative and analytical evaluation of properties. Several tables and diagrams were created to compare the properties of traditional polymer composites with microparticle fillers and nanomodified analogs. The main parameters compared were the modulus of elasticity, tensile strength, specific thermal conductivity, electrical conductivity, heat resistance, gas permeability, and wear resistance [19]. Absolute and relative changes (%), where possible, were given for all values to interpret the effect even at low nanofiller concentrations.

The fourth stage was morphological analysis and interfacial adhesion. Particular attention was paid to how the morphology of the nanoparticles (i.e., size, shape, state of aggregation, and surface functionalization) affected their uniform distribution in the polymer matrix, the nature of the bond at the interface, and the composites’ performance characteristics [20]. This analysis incorporated TEM/SEM micrographs from existing literature that confirm the effects of orientation, barrier action, and percolation structure formation.

A comprehensive assessment of the structure of polymer nanocomposites is increasingly based on a combination of several complementary analytical methods. For example, in addition to electron microscopy, X-ray diffraction (XRD) is widely used to analyze the degree of crystallinity and interlayer spacing in layered nanofillers. Raman mapping is used to assess the spatial uniformity of the distribution of carbon nanostructures; Raman mapping is used to assess the spatial homogeneity of the distribution of carbon nanostructures, nanoindentation is used to determine the local mechanical properties of the interphase layer, and WAXS and SAXS methods are used to study the supramolecular organization of the polymer matrix. X-ray microtomography (Micro-CT) is a promising tool for three-dimensional assessment of the internal structure, as it allows for the detection of porosity, agglomerates, and the spatial distribution of nanofillers without destroying the sample [7].

The fifth stage of the analysis involved a critical generalization using SWOT methodology [20,21]. A table was created to compare the strengths and weaknesses of PNCs and evaluate external opportunities, such as expanding applications and green technologies, and threats, including regulatory barriers, high costs, nanotoxicity, and instability over time. This review’s methodology ensures depth in interdisciplinary analysis and transparency in criteria selection, making it a template for similar studies of functional PNCs [15,22].

## 3. Results

### 3.1. Global Research Landscape and Market Trends

Several technologically and scientifically advanced countries are the world leaders in research and implementation of PNCs. These countries developed scientific infrastructure, powerful industrial clusters and high levels of investment in research and development. Table 1 provides a brief overview of their activities and features.

The United States and China retain leading positions in terms of publications and patents, while Europe and Japan demonstrate a more applied orientation, integrating PNC into industrial sectors. This confirms that leadership in nanocomposites is determined by the number of studies and the level of their commercialization. Italy, Canada, the United Kingdom, Brazil, and Israel also have specialized research programs or startup incubators in this area. According to Scopus and WIPO, from 2018 to 2024, China, the United States, and the EU accounted for more than 60% of the world’s patents and publications in nanocomposites, with China’s growth rate exceeding 12% per year [23].

### 3.2. Types and Properties of Nanofillers

**Figure 2:**
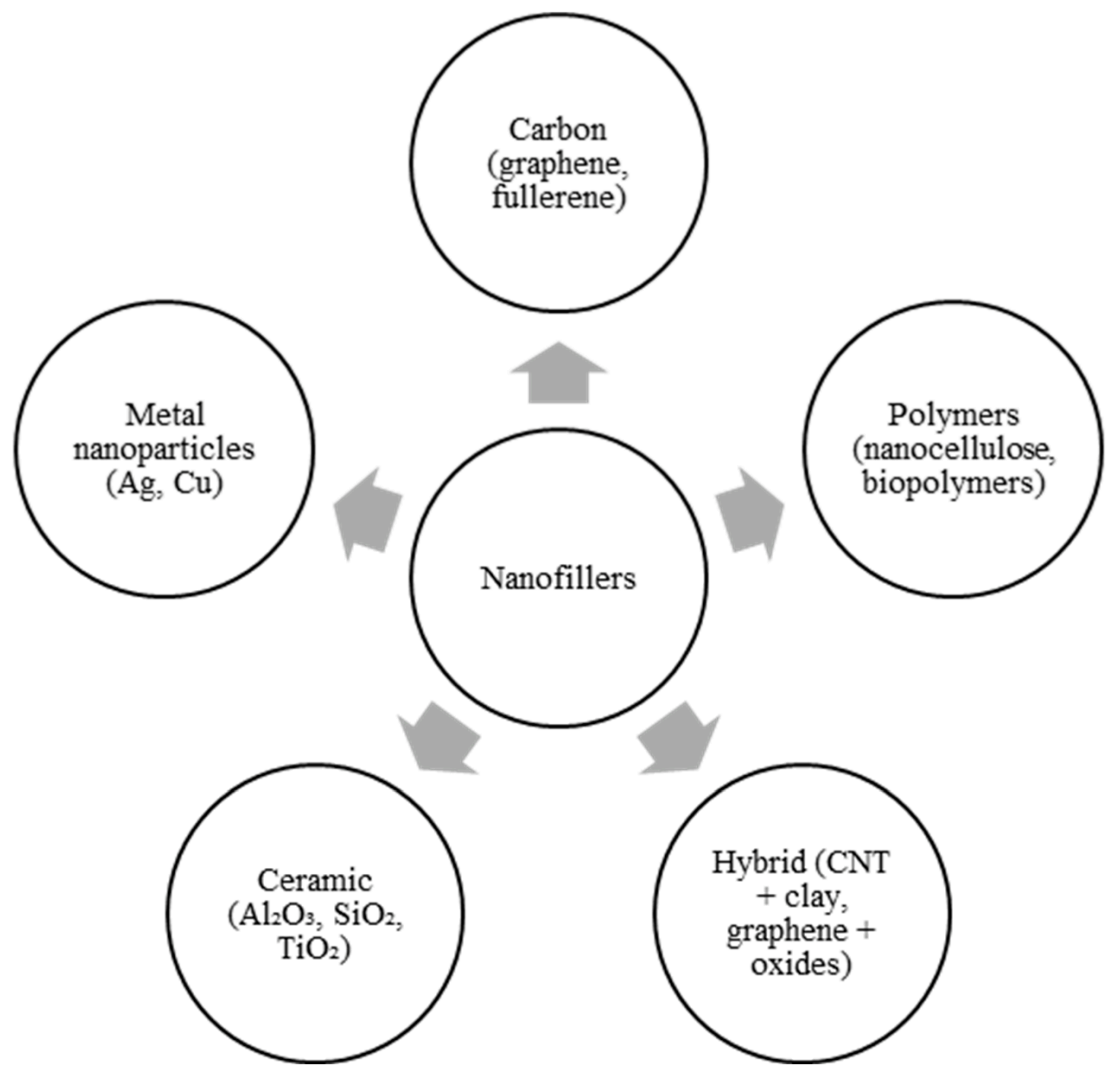
An original classification of nanofillers, developed based on an analysis of current literature for the purposes of discussion within this review article. Source: developed by the authors.

Depending on their nature, nanofillers in polymer composites are divided into several main groups: carbon, metal, ceramic, and polymer or hybrid nanostructures (Figure 2).

Carbon nanofillers include fullerenes (C_60_, C_70_ and higher), carbon nanotubes (hereinafter—CNTs) (single- and multi-walled, SWCNTs and MWCNTs), graphene, nanosheets, astralens, and carbon nanofibers [24]. These materials are characterized by ultra-high electrical conductivity, thermal stability, chemical inertness, and exceptional mechanical properties. For example, the modulus of elasticity of CNTs reaches 1.8 TPa, and the electrical conductivity exceeds 10^7^ S/m, which is hundreds of times higher than that of traditional conductive fillers [18,25].

Metallic nanofillers, such as silver, gold, copper, and nickel nanoparticles, can modify polymers to produce composites that are conductive, thermally conductive, or magnetically sensitive. Their unique properties arise from surface plasmon resonance, which intensifies their interaction with electromagnetic radiation [22,26]. These particles are mainly synthesized by chemical or physical methods, such as reduction in a colloidal solution, laser ablation, and ion implantation. The analysis systematized the main types of nanofillers, their effectiveness, applications, concentration thresholds, and risks. Table 2 provides consolidated information for comparing traditional and nanomodified fillers.

Thus, carbon nanofillers have the greatest potential for electronics and sensors due to their combination of strength and conductivity, while ceramic oxides prevail in areas where UV and wear resistance are critical. Ceramic nanofillers, which include metal oxides such as TiO_2_, SiO_2_, ZnO, Al_2_O_3_, and BaTiO_3_, provide high rigidity, thermal and UV resistance, and the ability to form electrets. They also improve the dielectric properties of polymers. Using such nanoparticles in polymer films, protective coatings, and photonic materials produces nanocomposites with high transparency, mechanical stability, and antibacterial properties [24]. Polymeric and hybrid nanofillers include dendrimers, functionalized polymers, and conductive structures based on doped polyanilines and polythiophenes. They also include multifunctional nanoparticles with an organic-inorganic nature. These nanofillers can self-organize to create nano- and mesoscale structures with enhanced adhesion and targeted functionality [17].

### 3.3. Comparative Performance of Traditional vs. Nanomodified Composites

Compared to traditional composites, nanomodified polymers demonstrate significant enhancements in mechanical properties with minimal filler content, typically less than 5 wt%. Polymer composite materials modified with traditional microfillers, such as glass fibers, talc, kaolin, graphite, and metal powders, are widely used to enhance the mechanical strength, stiffness, heat resistance, and fire resistance of base polymer matrices [28]. In such cases, the strengthening mechanism is based on mechanical reinforcement by inclusions with relatively large geometry and low specific surface area. However, the effectiveness of micronutrient fillers is limited at low concentrations. Achieving a significant enhancement of properties requires introducing 15–40 wt.%, which can lead to deterioration of technological characteristics, increased product weight, and decreased melt processability and viscosity [26].

In contrast, using nanofillers such as CNTs, graphene, nanofibers, nanoclay, and oxide or metal nanoparticles can significantly improve the performance characteristics of NCMs, even at concentrations not exceeding 0.5–5 wt%. Such efficiency is mainly due to the extremely high specific surface area of nanophases (up to 600 m^2^/g), the development of interfacial adhesion at the semi-solid polymer matrix interface, and the unique quantum mechanical and physicochemical properties at the nanoscale [18]. Consequently, these composites exhibit enhanced mechanical and thermal properties, as well as new functional properties such as electrical conductivity, magnetic sensitivity, and barrier properties. Table 3 compares the main characteristics of polymer composites with traditional fillers and nanofillers.

The comparative analysis in Table 3 confirms that nanofillers provide a significant increase in properties at minimal concentrations, which cannot be achieved by microadditives. This makes them effective for high-tech applications where the lightness and multifunctionality of materials are critical. For instance, in the case of adding only 1 wt.% of CNTs to an epoxy resin, an increase in the modulus of elasticity by about 20% and tensile strength by 25% is observed, which exceeds the efficiency of traditional microfillers, requiring at least 15–20 wt.% of fillers for similar enhancement. This effect is due to the strength of the nanotubes and their ability to transfer loads through interfacial bonds formed at the atomic level. The influence of nanophases on thermal characteristics is also significant. For example, the glass transition temperature (Tg) of polyamide-6 or epoxy resins can increase by 20–40 °C when 2–3 wt% nanoclay is added due to the restriction of macromolecular mobility in the interfacial interaction zone. In addition, owing to the percolation, when 0.1–2 wt.% of CNTs, graphene, or carbon ink is added, the electrical conductivity of the polymer composite can increase by 6–10 orders of magnitude [19,30].

### 3.4. Morphology, Interfacial Interactions, and Dispersion Effects

The morphology of nanoplatelet particles (nanoclay and graphene oxide) creates the so-called labyrinth effect, which complicates the diffusion of gas or vapor molecules. Consequently, the permeability of oxygen or water vapor may decrease by 80–90%, even at a dosage of 2–3 wt%. This is important for packaging materials and producing structural elements that require tightness. These data confirm the critical role of nanofillers in forming multifunctional polymer composites of a new generation. Nanofillers allow for the simultaneous improvement of multiple properties without increasing weight or deteriorating processability significantly, making such materials attractive for use in the aviation, automotive, electronics, and packaging industries. Table 4 summarizes the literature data and reflects the relative changes in the key characteristics of PNCs depending on the type of nanofiller and its concentration.

It should be noted that the ranges of variation in mechanical, thermal, and electrical properties presented in Table 4 are generalized based on the results of various independent studies, which differ in terms of the type of polymer matrix, the nature and concentration of nanofillers, the methods used for their functionalization, composite manufacturing technologies, and testing conditions. Therefore, the numerical values presented should not be regarded as a direct quantitative assessment of the effectiveness of individual types of nanofillers. They reflect characteristic trends in property changes established in the current scientific literature and are intended primarily for a comparative analysis of general patterns in the development of polymer nanocomposites.

**Figure 3:**
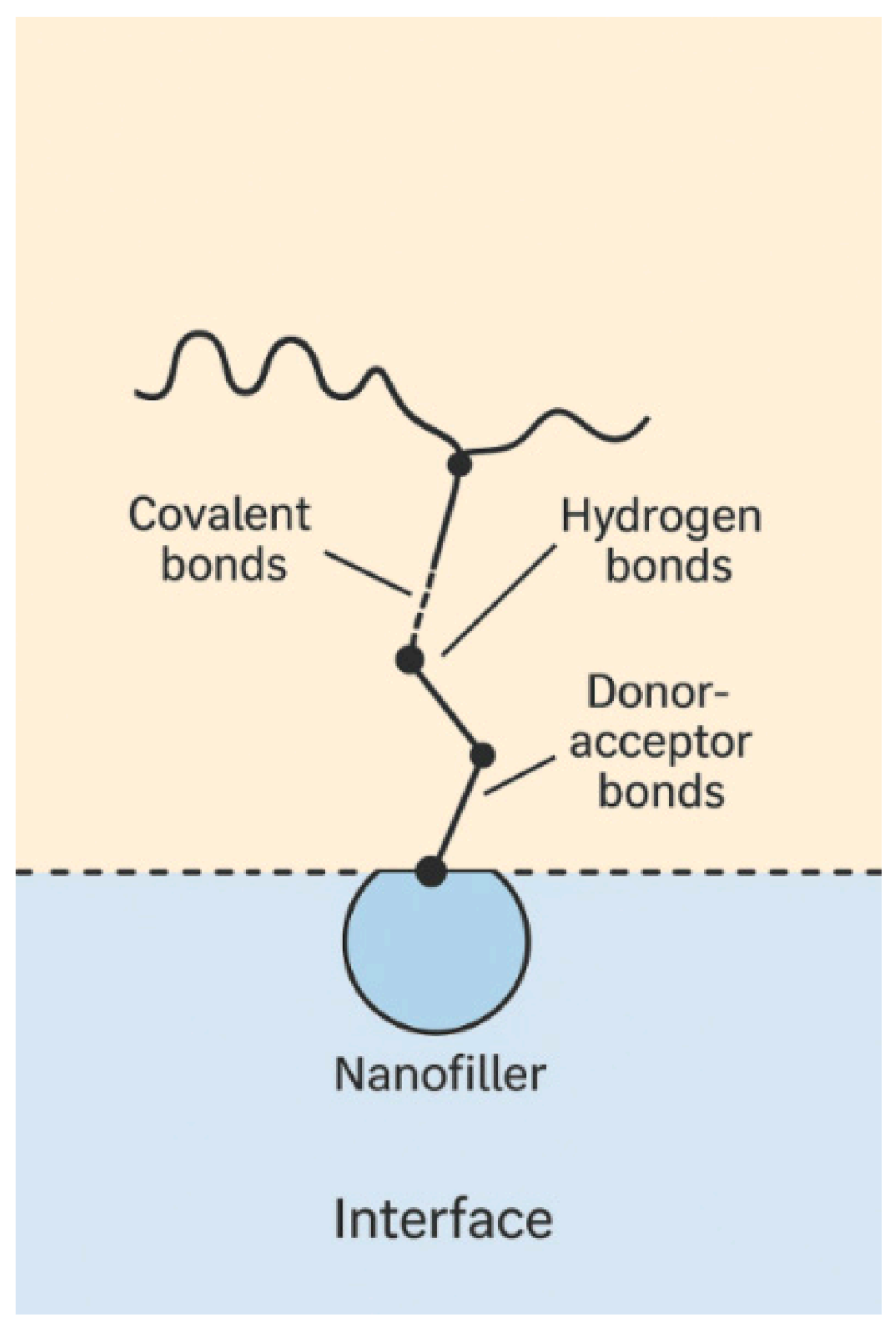
Schematic representation of the polymer-nanofiller interface with the formation of covalent, hydrogen, and donor-acceptor bonds. Scheme developed by the authors based on the interfacial interaction mechanisms described in [2,13,24].

Hybrid systems provide the highest balance between mechanical, thermal, and electrical properties, while single nanophases have a narrower specialization. This indicates the prospects of combined approaches. However, achieving the expected level of performance depends directly on the microstructure of the composite and, in particular, on how the nanofiller interacts with the polymer matrix. The morphology of the nanofiller is one of the key factors that determines the effectiveness of a polymer nanocomposite. This includes parameters such as particle size, shape, degree of aggregation, and chemical surface functionalization. These characteristics directly affect the uniformity of nanoparticle distribution in the polymer matrix and interfacial adhesion. They are determining factors for the creation of an effective mechanical bond between the organic matrix and the inorganic nanophase. The mechanisms of interfacial interaction are shown in Figure 3.

Another important aspect is to confirm the existence and nature of interfacial interactions. In particular, X-ray photoelectron spectroscopy (XPS) records changes in the chemical states of surface atoms after modification or integration into the polymer, indicating the formation of covalent or coordination bonds. Infrared spectroscopy (FTIR) allows for detecting shifts in the characteristic peaks of functional groups caused by the formation of hydrogen or donor-acceptor bonds at the interface. For carbon nanofillers, the key indicator is the change in the ratio of the intensities of the D and G peaks in the Raman spectra, which reflects the modification of the structure of graphene layers or nanotubes in the presence of a polymeric environment. The combination of these methods forms a reliable evidence base that confirms the formation of interfacial contacts and explains their effect on the mechanical, electrical, and barrier properties of PNCs.

The size of the nanofiller significantly impacts the specific surface area. The smaller the particles, the greater the interaction area with the polymer. However, small particles are prone to aggregation, making it difficult to disperse them evenly in the matrix. Aggregate formation leads to local inhomogeneities and structural defects that reduce the composite performance characteristics, particularly its mechanical and impact strength. To avoid this, it is advisable to use intensive dispersion methods, such as ultrasound or high-shear mixing, or to modify the surface of the nanoparticles to reduce their ability to adhere. In addition, particle shape plays an equally important role in structure formation. Needle-shaped nanofillers, such as CNTs, can form percolation structures that improve the material’s electrical conductivity significantly. Lamellar fillers, such as graphene or montmorillonite nanoclays, form barrier layers in the polymer that block moisture and gas penetration. Despite their lower ability to orient, spherical nanoparticles provide isotropic modification of properties, which is useful in a number of functional applications.

**Figure 4:**
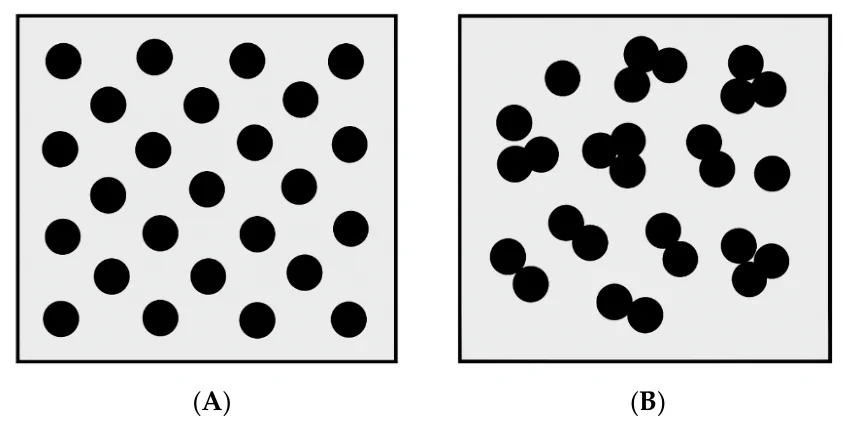
Schematic representation of the distribution of nanoparticles in the polymer matrix: (**A**) uniform dispersion of the nanophase, which provides high interfacial adhesion and improved composite properties; (**B**) aggregation of nanoparticles with the formation of agglomerates, which leads to structural defects and degradation of performance characteristics. Scheme developed by the authors based on [2,27,30].

The aggregate state of the system and the tendency of nanoparticles to aggregate are critical for structural homogeneity and the stability of properties over time. If the nanofiller dispersion is not sufficiently stabilized, agglomerates are formed (Figure 4B), which impair interfacial interaction and cause structural defects. In contrast, the uniform distribution of nanoparticles in the polymer matrix (Figure 4A) ensures the formation of a stable microstructure with a high specific area of phase contact, which contributes to more efficient load transfer, improved mechanical characteristics, and increased barrier and electrical properties of the composite. Thus, controlling dispersion and preventing aggregation are crucial factors for realizing the potential of nanofillers in polymer systems.

To prevent these effects, the surface functionalization of nanoparticles with the introduction of active groups (carboxyl, amino, silane, etc.) is widely used, which increases their compatibility with the polymer phase and promotes uniform distribution. Dispersion homogeneity is a key factor in realizing the properties of the composite, so in the analyzed works both structural and physicochemical methods are used to evaluate it. Transmission electron microscopy (TEM) allows visualization of agglomerates and determination of their average size, while small-angle X-ray scattering (SAXS) provides information on the characteristic distances between nanoparticles and the correlation length, which serves as a quantitative indicator of dispersion. In addition, the rheological analysis is used, where the appearance of a plateau in the shear modulus G′ at low frequencies confirms the formation of a percolation network. In some studies, atomic force microscopy (AFM) is used to show the surface topography and spatial organization of nanofillers in the polymer matrix.

Surface functionalization is an important tool for improving interfacial adhesion. It enables the formation of specific interactions between the matrix and the nanofiller, resulting in chemical or physicochemical bonds at the interface. This connection improves the transfer of mechanical load from the polymer to the nanophase. This transfer is a prerequisite for increasing the material’s strength, stiffness, and durability. For instance, functionalized CNTs interact more effectively with polar polymer matrices, such as polyamide or epoxy resins, than non-functionalized CNTs. This results in a higher modulus of elasticity and better fracture resistance [30].

Mechanical load transfer in polymer nanocomposites occurs through an interphase layer that forms at the interface between the polymer matrix and the nanofiller. This region has physicochemical properties that may differ significantly from those of both the polymer matrix and the nanofiller itself. The thickness of the interfacial layer depends on the nature of the components, the degree of surface functionalization, the molecular mobility of the polymer chains, and the conditions under which the composite is manufactured. It is the interphase layer that ensures the effective transfer of stresses from the less rigid polymer matrix to the high-strength nanofiller, which determines the level of mechanical reinforcement of the material. Insufficient adhesion or the presence of defects in the interfacial zone significantly reduces the efficiency of load transfer, which can lead to the premature initiation of microcracks and the failure of the composite even when the nanofiller itself has high intrinsic properties.

### 3.5. Processing Methods, Applications and Case Studies

The morphological parameters of the nanofiller are also determining factors that influence the functionality, stability, and technological suitability of a polymer nanocomposite. To explain the mechanisms by which the morphology of nanofillers influences the mechanical, electrical, and barrier properties of polymer nanocomposites, analytical models—including the Halpin–Tsai and Mori–Tanaka models, as well as percolation theory—are widely used in current research. The Halpin–Tsai model is used to predict changes in the elastic modulus of composites as a function of geometric parameters and nanofiller concentration, while the Mori–Tanaka model is used to evaluate the effective mechanical properties of heterogeneous materials, taking into account the interaction between the matrix and the inclusions, while percolation theory describes the formation of continuous conductive networks and a sharp increase in electrical conductivity upon reaching a critical concentration of nanofillers. At the same time, most existing models are based on simplified assumptions regarding uniform particle distribution and ideal interfacial adhesion; therefore, they do not fully account for agglomeration, structural anisotropy, heterogeneity of the interfacial layer, and the technological features of polymer nanocomposite fabrication. For this reason, the results of mathematical modeling should be considered in conjunction with experimental studies of the structure and properties of materials.

It should be noted that the performance characteristics of polymer nanocomposites are determined not only by the nature of the nanofiller but also by the complex interaction of a number of interrelated factors. These include the volume or mass fraction of the nanofiller, particle size and shape, aspect ratio, degree of dispersion in the polymer matrix, the effectiveness of interfacial adhesion, and the presence of surface functionalization, as well as the manufacturing technology and processing conditions of the material. Even for identical types of nanofillers, a change in even one of these parameters can significantly affect load-transfer mechanisms, the formation of percolation structures, the development of residual stresses, and the final mechanical, electrical, and barrier properties of the composite. For this reason, it is advisable to evaluate the effectiveness of various nanofillers only by considering a combination of technological and structural factors, rather than solely based on the type of nanophase used.

However, the effectiveness of PNCs is also largely determined by the method chosen for integrating them into the polymer matrix. The method by which the nanophase is incorporated determines its level of dispersion, degree of aggregation, and interfacial adhesion. These factors affect the composite’s entire range of mechanical, heat-resistant, electrical, and barrier properties. Therefore, modern approaches to manufacturing nanocomposites fall into three main categories: solvent methods, melt mixing, and in situ synthesis. In addition, methods of surface functionalization of nanofillers have become widely used to ensure their compatibility with the polymer matrix at the molecular level.

Dissolution technology is based on the ability of polymers and nanofillers to disperse evenly in a liquid medium that later evaporates, forming a solid composition. In this process, nanoparticles are dispersed in a solvent along with a polymer or monomer to ensure the formation of a uniform nanocomposite layer once the solvent has evaporated. This approach is commonly used to synthesize nanocomposites based on epoxy resins, polyamides, polyesters, and polyimides because it can produce heat-resistant, transparent, and flexible structures [30]. However, limitations include the use of toxic and volatile organic solvents, the complexity of disposal, and the difficulty of completely removing solvent residues to avoid defects in the matrix. Technological difficulties include high energy consumption, long drying times, and sensitivity to environmental conditions (Table 5).

As can be seen from Table 5, each approach has advantages and limitations. Solvent-based methods provide high-quality dispersion and uniformity of structure. However, they are characterized by increased energy consumption, high costs at the stage of evaporation and solvent recovery, and environmental risks, which complicates scaling. On the other hand, mechanical mixing in the melt is the most economical and industrially applicable method, as it does not require organic solvents and can be implemented on standard extrusion equipment. Nevertheless, it is less effective in ensuring stable interfacial interaction. Polymerization provides a high level of dispersion and the formation of strong interfacial bonds, although it is inferior in terms of ease of execution, requires careful control of reaction conditions, and is currently less scalable for industrial production. Thus, the choice of method is determined by a compromise between the quality of the structure, energy consumption, and economic feasibility.

Melt blending is one of the most widely used industrial methods for manufacturing nanocomposites. This is due in part to its ease of implementation, high productivity, and scalability. This process involves mixing a molten polymer with nanofillers using extruders, rollers, or screw mixers. After intensive mixing, the material is cooled and either granulated or molded directly into a product. However, the effectiveness depends significantly on the ability to disaggregate nanophases and the strength of the interfacial interaction between the polymer matrix and the particles. Due to their high specific surface area and interparticle van der Waals forces, nanoparticles often aggregate, forming agglomerates that impair the homogeneity of the structure. In order to eliminate these effects, ultrasonic dispersion, plasticization, the introduction of dispersants, and the surface modification of nanofillers to improve compatibility are used. This approach allows for the avoidance of solvents and the processing of a wide range of polymers, including thermoplastics and thermosetting systems [13].

In situ synthesis involves polymerization in the direct presence of nanofillers, which serve as either substrates or initiators of polymer chain growth. Typically, the nanoparticles are pre-dispersed in a monomeric medium before polymerization occurs to form a composite. This approach enables a higher degree of intercalation or exfoliation of layered nanophases (e.g., montmorillonites, graphene oxides, and nitrides), significantly improving the material barrier and mechanical properties [32]. For instance, polymerizing ε-caprolactam with modified clay nanoparticles or functionalized CNTs creates structures with directed interfacial bonds, increasing the strength and heat resistance of polyamide. A significant advantage of this method is the chemical fixation of nanophases within the matrix, enhancing structural stability and reducing particle migration [13].

Chemical surface functionalization of nanofillers is critical to ensuring interfacial adhesion and improving dispersion in hydrophobic or hydrophilic polymeric media. The surface of most inorganic nanoparticles, such as SiO_2_, Al_2_O_3_, or TiO_2_, is hydrophilic, which prevents them from mixing uniformly with polyolefins or other nonpolar polymers. To eliminate this issue, the surfaces are modified with functional groups through processes such as silanization, halogenation, and the grafting of amino or epoxy groups. Addition reactions with isocyanates are also employed. For instance, introducing amino groups onto silica nanoparticle surfaces enables the formation of covalent bonds with epoxy matrices, increasing shear strength by 30–50%. Using modified CNTs with carboxyl or hydroxyl groups improves dispersion in polyester and polyamide while reducing the likelihood of agglomeration.

Thus, choosing the right synthesis method and integrating nanophases are critical to achieving a stable structure and optimal properties in polymer nanocomposites. Modern research focuses on combining methods, such as the hybrid approach of in situ polymerization with pre-functionalization or the mechanical dispersion of nanophases in solution. This opens new avenues for the controlled formation of nanostructured polymeric materials with predictable properties. These approaches are already being implemented in several industries (Table 6), where PNCs improve the performance of materials.

Table 6 shows that in each industry, the key factor is specific functionality: barriers in construction, strength with low weight in aviation, antibacterial in medicine, transparency and flexibility in electronics. This emphasizes the adaptability of PNC to various technological requirements. In construction, PNCs are used as structural and protective materials. For example, adding nanoclay to a polymer matrix can greatly enhance its barrier properties against moisture and gases while increasing fire resistance by forming a protective, heat-resistant layer under thermal load. This reduces the need for traditional flame retardants, many of which are toxic or environmentally hazardous. In addition, polymer coatings with nanophases demonstrate increased abrasion resistance, making them ideal for protecting metal structures, facades, and industrial floors. Nanoporous composites that combine low thermal conductivity and mechanical strength are used in thermal insulation systems.

In the aviation industry, PNCs are used to manufacture structural elements such as the skin, stabilizers, wings, and sealing elements, where strength and weight are critical factors. Epoxy matrices modified with CNTs allow for increased impact resistance without increasing weight significantly, which is crucial for fuel efficiency. In the automotive industry, nanocomposites are used to produce interior parts and electronic components. For instance, Noryl GTX, a material containing functionalized nanotubes, is used to produce lightweight, antistatic mirror and bumper housings that demonstrate improved electrical conductivity and resistance to environmental influences.

In healthcare, nanofilled polymers are used to create bioinert implants, antibacterial coatings, and microcapsules for targeted drug delivery. They are also used as functional elements in diagnostic systems. Adding silver nanoparticles or zinc oxides to polymer matrices creates an antibacterial effect that was confirmed by several in vitro studies. Membranes that incorporate fullerenes or graphene oxide enable precise control of permeability to specific biomolecules. This is crucial for selective filtration and transporting drugs into cellular media. Biocompatible nanocomposites such as polylactide or polycaprolactone with nanohydroxyapatite promote bone regeneration during orthopedic and dental procedures [33].

PNCs open up new possibilities for electronics and sensor technologies due to their combination of flexibility, electrical conductivity, and transparency. Polymers containing graphene, graphene oxide, and CNTs are particularly promising because they provide electrical conductivity while maintaining the optical transparency and elasticity necessary for creating flexible displays, smart textile elements, and flexible temperature, strain, and biometric sensors. These nanocomposites form the basis for thin-film sensors, RFID tags, and self-healing, multifunctional surfaces in wearable electronics. Important parameters for such applications are dielectric loss and breakdown strength, which determine the reliability of operation under high stress and variable frequencies. The optimal introduction of nanoparticles (SiO_2_, Al_2_O_3_, functionalized graphene) can reduce dielectric losses by 15–30% in a wide frequency range and increase the breakdown strength by 20–40%. This affects the durability and safety of electronic devices directly [15,21]. As a result, PNCs become the basis for the creation of thin-film sensors, RFID tags, and self-healing and multifunctional surfaces in wearable electronics.

### 3.6. Technological and Industrial Challenges

Despite significant advances in the development and application of PNCs, several fundamental and applied issues still prevent their widespread industrial implementation [29]. The tendency of nanoparticles to aggregate in a polymeric environment is one of the key technological obstacles. It limits the efficiency of their distribution significantly and reduces the intensity of interfacial interaction. Aggregate formation worsens the mechanical and barrier properties of the composite and complicates processing and injection molding. Moreover, the effective concentration of nanofillers is limited and usually does not exceed 5 wt.%. Exceeding this threshold often results in a loss of rheological stability of the melt, increased viscosity, processing difficulties, and a heterogeneous structure in the finished product. These limitations require improvements in methods for functionalizing the surface of nanoparticles and determining optimal phase ratios.

Another essential aspect is the long-term stability of nanofillers in a polymer environment. When exposed to moisture, temperature fluctuations, or ultraviolet radiation, the functional properties of composites degrade over time. From an economic standpoint, the widespread use of expensive nanofillers, such as fullerenes, graphene, and CNTs, is financially unfeasible for many industries. This is particularly relevant for mass production, where even a slight increase in material cost can be critical. Developing scalable synthesis technologies and improving processing are key to overcoming this barrier. The bioethical and environmental risks associated with the use of nanoparticles deserve special attention. The potential toxicity of nanomaterials, their impact on living organisms, and the mechanisms by which composites decompose or are recycled are subject to active international monitoring. There is an urgent need to standardize toxicological analysis methods, establish a regulatory framework for managing wastes, and develop environmentally friendly alternatives.

The literature also reports the effect of long-term aging conditions, such as UV irradiation and humidity cycles, on the performance of PNCs [2]. Such loads are accompanied by a gradual degradation of mechanical properties and a decrease in interfacial adhesion. Microscopic studies (SEM/TEM) in a number of works document the characteristic mechanisms of fracture after aging—delamination at the interface, formation of microcracks, and tearing of nanoparticles from the matrix [27]. In addition, in the case of nanofillers based on metals and oxides, ion leaching phenomena are reported, which is associated with potential toxicological risks [13,14]. These results emphasize the need for a long-term assessment of the stability and safety of nanocomposites in real-world applications.

### 3.7. Drawbacks and Future Perspectives

Despite these challenges, the development of PNCs remains positive. Intensive research of self-organization of nanostructures, functionalized graphene hybrids, 3D printing of composites with oriented nanofillers, and the introduction of scalable synthesis methods using available precursors forecasts a significant expansion of PNCs’ scope in the coming decades [14]. Table 7 shows the main functional effects, minimum effective concentrations, typical applications, main risks of implementation, and estimated cost.

Table 7 concludes that the trade-off between efficiency, cost, and environmental risks is crucial when choosing nanofillers. In particular, carbon systems are the most efficient functionally, but their cost and toxicity remain a barrier to mass adoption.

## 4. Discussion

Using nanofillers in polymer matrices creates new opportunities to modify material properties, surpassing the capabilities of traditional micro- or macro-fillers. One advantage of nanoscale structures is their high surface-to-volume ratio, which increases the area of interfacial contact between the filler and polymer. Along with the effects of quantum confinement and nanoscale interactions, this improves the performance of the composite, even at low nanophase concentrations [13,36].

The mechanical characteristics of the polymer matrix can be improved by 20–50% due to the uniform distribution of nanoparticles, which block the local movement of macromolecules under load. Unlike traditional micronutrient fillers, nanoparticles do not generate stress concentrators. Instead, they create a more homogeneous energy field within the material. The effectiveness of the reinforcement correlates directly with the quality of interfacial adhesion, which maximizes due to the high specific surface energy [2,27]. At the same time, there are contradictory data in the literature. Some studies [12] indicate an increase in brittleness when the nanophase concentration exceeds a certain threshold. This emphasizes the importance of optimizing the concentration ranges for each class of nanofillers.

In addition, nanofillers can increase the thermal stability of composites. This is achieved by restricting the mobility of polymer chains near the surface of nanophases (the so-called trapped layer effect), which increases the glass transition temperature (Tg) and stabilizes the structure at elevated temperatures. These changes are relevant for structural materials operating under thermal stress [35]. However, the literature is inconsistent. Thus, a significant increase in Tg is recorded for amorphous polymers, while the results remain inconsistent for semicrystalline systems [34]. Another important aspect is the formation of electrically conductive and magnetically sensitive nanostructures. Using CNTs, graphene, or metal nanoparticles facilitates the creation of composites that can conduct electric current when the percolation threshold is reached, even at concentrations < 1 wt. % (Table 3). This creates opportunities for creating antistatic coatings, electromagnetic shielding, and flexible electronics [14,32].

The barrier and diffusion properties of polymers are also improved significantly due to the labyrinthine diffusion effect that occurs when nanoplatelet fillers (e.g., clay, graphene) are arranged in an orderly fashion. This configuration hinders the penetration of gases and water vapor through the material, making PNCs effective for packaging, membrane systems, and coatings [24]. In addition to these functional advantages, replacing heavy fillers with nanostructures that have a much lower density reduces the weight of finished products. Combined with high mechanical efficiency, this ensures the optimization of structures without strength loss, which is a critical factor for the aerospace, transportation, and energy industries [6,27,37]. However, the influence of the spatial orientation of nanoplatelet structures on the gas permeability of polymers is described only qualitatively, without a systematic quantitative analysis, which is a significant gap in research.

Nanoparticle surface functionalization facilitates the control of interfacial interactions and the embedding of new properties into materials, including antibacteriality, UV resistance, and biocompatibility. These properties create a new generation of adaptive materials that can respond to external influences or integrate with biological environments. Thus, nanofillers greatly expand the range of functionality of polymeric materials, offering the chance to produce multifunctional composites with enhanced performance characteristics [11,14]. Although there are obvious advantages of using nanofillers to create polymer composites, their widespread use presents significant technical and socio-environmental challenges. One disadvantage is the aggregation of nanoparticles due to their high specific surface energy. Consequently, agglomerates form, worsening the uniformity of nanophase distribution in the polymer matrix, reducing interfacial adhesion, and negating the expected improvement in composite properties. This is particularly critical when working with CNTs, graphene, or metal nanoparticles, where effective dispersion is essential for achieving the desired outcome [34,38].

At the same time, the spatial orientation of nanofillers does not always have an exclusively positive effect. For nanofillers with a high aspect ratio, such as carbon nanotubes and graphene nanosheets, orientation along the flow direction of the polymer melt is characteristic during extrusion or injection molding. This results in an anisotropic material structure, leading to differences in mechanical, thermal, and electrical properties between the longitudinal and transverse directions. Therefore, when designing structural products, it is necessary to consider the potential impact of processing technology on the spatial distribution of nanofillers and the associated anisotropy of properties. In addition to agglomeration, air pores, microvoids, and defects in the surface functionalization of nanofillers negatively affect the performance characteristics of polymer nanocomposites. Such inhomogeneities act as local stress concentrators, promote the initiation and propagation of microcracks, and can significantly reduce the material’s durability even when static strength values are high. Minimizing the number of such defects is one of the key objectives in improving the manufacturing technologies for polymer nanocomposites.

Another important factor is the technological complexity of implementing nanomodification in mass production. Mixing, in situ polymerization, dispersion, and extrusion require careful control of temperature, shear rate, and system viscosity [13]. Adapting the equipment or using ultrasonic, hydrodynamic, or high-temperature methods increases energy consumption and the cost of the final product [35]. Furthermore, technical personnel require special training, which complicates the integration of nanotechnology into traditional production lines. The stability of nanocomposites over time is also a matter of debate. Some nanofillers, especially those with exposed metal surfaces or unstable functional groups, are susceptible to oxidation, aggregation, or chemical degradation in environments with high humidity or UV radiation. Such destruction can reduce the material functionality and lead to unexpected changes in its properties over time [14,33].

An analysis of the current literature indicates that the assessment of the prospects for the practical application of polymer nanocomposites should not be limited solely to the results of static mechanical tests. Dynamic mechanical analysis (DMA) is increasingly being used for a comprehensive assessment of operational reliability; it allows for the determination of the storage modulus, loss modulus, and glass transition temperature based on changes in the tan δ parameter. In addition, tests for fatigue, crack resistance, wear, and erosion resistance are of great importance, as they provide a more complete understanding of material behavior under long-term service conditions.

The economic aspect is also critical. While the cost of nanomaterials is gradually decreasing due to increased production, the cost of CNTs, fullerenes, graphene, and functionalized metal oxides remains higher than that of conventional fillers. This renders nanomodified composites financially infeasible for many industries with limited budgets, including consumer electronics, packaging, and the mass construction sector [15,25]. Moreover, the absence of globally recognized standards for assessing the toxicity of nanoparticles, the lack of long-term epidemiological studies, and the difficulty of controlling the emission and disposal of nanomaterials create significant bioethical and environmental barriers. The ability of nanoparticles to penetrate biological membranes and accumulate in the body is concerning to the scientific community and regulatory authorities.

Meanwhile, there are virtually no systematic LCA studies that cover the entire life cycle of nanocomposites. This limits the possibility of an objective assessment of their real environmental footprint and comparison with traditional materials. In this regard, systematic toxicological studies, standardization of procedures for handling nanomaterials, and the creation of a legislative framework for their safe use are needed [11,36]. To systematize the identified problems and ways to overcome them, the technological and industrial challenges in the manufacture of PNCs are summarized in Table 8.

Due to the rapid growth in interest in functional nanostructures and the increasing demand for material properties in key industries such as electronics, medicine, energy, and aviation, approaches to the development and production of PNCs are gradually transforming. Analyzing current publications, market analytics, and technological roadmaps permits the identification of several strategic development vectors. The primary condition for reducing PNC costs is transitioning from laboratory to industrial-scale synthesis of nanofillers such as graphene, CNTs, hybrid nanoclay, and nanocellulose fibers. Thermochemical exfoliation of graphite and hydrothermal synthesis enable the production of large quantities of stable nanomaterials at lower costs.

Furthermore, combining several types of nanofillers (e.g., metal/carbon, clay/biopolymer, and oxides/graphene) helps expand the material functionality while compensating for the individual components’ shortcomings. These hybrids provide an optimal balance between mechanical, electrical, and barrier properties. For example, CNT/Ag and Fe_3_O_4_/graphene–polymer composites have improved electrical conductivity, heat resistance, and antibacterial properties [31]. Moreover, there is growing interest in natural nanofillers such as nanocrystalline cellulose, hectorite or montmorillonite clay layers, and functionalized biopolymers. Using these materials aligns with the principles of green chemistry, reduces the ecological footprint of production, and enables the creation of biodegradable or bioinert composites. For instance, biopolymers modified with nano Ag are used in packaging and medicine without harming the environment.

In addition, integrating machine learning, molecular modeling, and computational chemistry methods into NCM design facilitates predicting composite properties at the component selection stage. Materials informatics and digital twins optimize composition, predict interfacial adhesion, and model structural and functional properties. These tools reduce the duration of the research-prototype-test cycle [29]. Using NCMs is expected to expand significantly into new niches, including smart packaging that responds to temperature or microbial contamination, energy-saving nanofilms for windows that can control light and heat transmission, membranes with nanopores for water purification or mixture separation, and nanofibers in functional textiles with thermoregulation or sensing properties [12].

An analysis of current research shows that the further development of polymer nanocomposites will involve the combination of multifunctional nanofillers, the refinement of dispersion technologies, digital modeling of material structure, and the use of artificial intelligence methods to optimize composite composition. Significant attention is also being paid to the creation of environmentally safe and recyclable polymer nanocomposites, in line with modern principles of sustainable development [7].

Therefore, the PNC development is focused on functionalization, digitalization, and sustainable development. Scientific and technological barriers are gradually being overcome through interdisciplinary integration and global collaborations in the fields of materials science, nanotechnology, and chemical engineering. Meanwhile, the current disadvantages of polymer nanocomposites (the high cost of nanofillers, the difficulty of ensuring a stable dispersion, technological difficulties in scaling up, and environmental and safety restrictions) remain significant challenges. They define the key areas of future research and stimulate the search for new solutions. A combination of innovations in functionalization, the creation of hybrid nanostructures, and the introduction of digital forecasting methods and green chemistry principles is expected to ensure the transition from experimental prototypes to widespread industrial use. As a result, polymer nanocomposites are poised to become the basis of the next generation of engineering materials by 2030 [33,34].

## 5. Conclusions

The study concluded that PNCs are among the most dynamic and innovative fields in modern materials science. By combining advances in nanotechnology, polymer chemistry, surface physics, and composite design, PNCs help design materials with specific characteristics. Nanofillers can alter the properties of the polymer matrix at low concentrations, minimizing the cost of raw materials while maintaining or improving the final product characteristics. The analysis of nanofiller types revealed that each class specializes in specific functional applications. For example, graphene, CNTs, and fullerenes provide the best electrical conductivity, strength, and thermal conductivity, while nanoclay, silica, and metal oxide nanoparticles improve barrier properties, fire resistance, and optical activity. These materials enable the development of a new generation of materials for use in aviation equipment, medical implants, biocompatible sensors, and adaptive structures.

Issues related to interfacial interactions and the aggregation of nanofillers are critical to ensuring the stability and effectiveness of the material. As the review showed, controlling the filler morphology, degree of dispersion in the polymer, and interface engineering are key to achieving the desired performance properties. Chemical functionalization, plasma surface treatment, and the use of compatible binders and modifiers are important in nanocomposite engineering. Developing hybrid nanofillers that combine functions from different classes (e.g., combining the electrical conductivity of carbon components with the photocatalytic activity of TiO_2_) proved promising. These solutions allow for a synergistic effect that cannot be achieved using a single type of nanomaterial. However, it was also determined that ensuring their chemical compatibility and controlled interaction with the matrix is necessary.

However, there were a number of constraints to the widespread introduction of NCMs into industry. These included the high cost of high-quality nanomaterials, difficulty scaling up laboratory technologies to industrial volumes, insufficient standardization, and lack of understanding of the long-term effects of using nanoparticles in household or biomedical applications. Moreover, serious concerns exist about the toxicity of nanostructures, their accumulation in ecosystems, and the difficulty of utilizing or recycling materials with embedded nanocomponents. Nevertheless, there is an increase in studies of green nanofillers, particularly nanocellulose, natural nanoglues, and biooxides, which have great potential for bio-based applications. Developing biocompatible compositions helps combine engineering efficiency with environmental responsibility to move to a circular economy.

Implementing digital technologies in the modeling and optimization of nanocomposites is important. Mathematical modeling, interfacial interaction simulations, and artificial intelligence help create knowledge bases that predict the properties of future composites before the synthesis stage begins. These advances shorten development time, lower costs, and improve the reliability of end products. According to current global market dynamics, China, the United States, Germany, Japan, and South Korea are leading producers and patent holders of polymer nanocomposite technologies. These countries invest heavily in nanotechnology infrastructure to support the development of cluster centers and startups and encourage interdisciplinary educational programs. This approach enables the creation of full-fledged technological ecosystems.

Therefore, the future of the PNCs industry hinges on improving material properties and integrating into sustainable production cycles, meeting modern environmental requirements, and interacting with digital platforms. Overcoming existing limitations requires coordination between academia, industry, regulators, and educational institutions for technological advancements in the next decade.

## Figures and Tables

**Table 1:** Comparison in the field of PNCs implementation.

Country	Key Research Directions	Features	Source
USA	Functional nanofillers (carbon nanotubes, graphene), biocompatible composites, nanomembranes	Leader in the number of patents and publications in the field of NCM; active commercialization of technologies	[23,24]
China	Mass production of nanomaterials, chemical modification of polymers	Highest growth rates in research and publications; dominance in the CNT and graphene market	[18,23]
Germany	Industrial PNCs, nanocomposites for the automotive and energy industries	Integration into the automotive and chemical sectors; support from government programs	[22,24]
Japan	Elastomers with nanophases, nanofillers for electronics, bio-oriented PNCs	High level of quality control, focus on electronics and medicine	[22,24]
South Korea	Smart nanocomposites, sensors, 3D printing	Strong integration with the IT sector, focus on materials for flexible electronics	[8]
France	Barrier films, biodegradable composites	Orientation towards eco-friendly solutions and new generation packaging	[11]
India	Low-cost nanocomposites based on natural polymers	Local needs and scalable low-cost technologies	[24]

**Table 2:** Types of nanofillers and their main properties.

Type of Nanofiller	Examples	Main Properties	Areas of Application	Sources
Carbon	C_60_, CNT, graphene	High strength, electrical conductivity	Aviation, electronics, biomedicine	[24,27]
Metal	Ag, Au, Cu	Conductivity, antibacterial properties	Electronics, sensors, pharmacy	[22,26]
Ceramic	TiO_2_, SiO_2_, ZnO	UV resistance, hardness, chemical inertness	Coatings, optics, filtration	[22,26]
Polymer/hybrid	Dendrimers, polythene	Self-organization, electrical conductivity	Medicine, intellectual materials	[17,24]

**Table 3:** Comparison of properties of traditional and nanomodified polymer composites.

Property	Traditional Microfillers (Glass, Talc, Graphite)	Nanofillers (CNTs, Graphene, Nanoclay)	Sources
Typical filler concentration	15–40 masses%	0.5–5 masses%	[2,22,28]
Specific surface area	<10 m^2^/g	дo 600 m^2^/g	[2,27]
Mechanical strength	Grows, but requires high dosage	Increases by 20–50% with dosing < 2%	[2,9,27]
	Tensile strength:		[2,27]
	Epoxy resin + 5 wt.% talc—growth by 10–15%	Epoxy resin + 1 wt.% CNT—growth by 25%	
	Modulus of elasticity		[2,24]
	PET + 5 wt.% kaolin—12% growth.	PET + 2 wt.% alumina nanoplates—growth by 25%	
Glass transition temperature (Tg)	Minor changes	Increase by 20–40 °C	[2,22]
	Polyamide-6 + 10 wt.% fiberglass—increase by 10–15 °C	Polyamide-6 + 3 wt.% nanoclay—increase by 30–40 °C	
Electrical conductivity	Remains low or does not change	It increases by 6–10 orders of magnitude when the percolation threshold is reached	[2,18,27]
	Polystyrene + 10 wt.% graphite—increase by 1 order of magnitude	Polystyrene + 0.5 wt.% CNT—growth by 6 orders of magnitude	
Barrier properties	Moderate, depending on the morphology of the filler	Up to 70–80% improvement due to the “maze effect”	[2,24,29]
	PET + 4 wt.% talcum powder—20% reduction in permeability	PET + 1 wt.% graphene wafers—permeability reduction by 80%	
Material density	Often increases due to the high weight of the filler	Minimal increase; even weight reduction is possible	[2,28]
Matrix compatibility	Depends on surface energy, often limited	Can be optimized by surface functionalization	[2,22]
Cost and availability	Low, well-developed infrastructure	Higher cost; limited industrial implementation	[2,24]
Processability	Well-known techniques	They require special methods of dispersion or in situ synthesis	[2,17,22]

**Table 4:** Effect of different types of nanofillers on the properties of polymer composites.

Nanofiller Type	Concentration (wt.%)	Mechanical Properties (%Δ Modulus of Elasticity/Strength)	Thermal Conductivity (%Δ)	Electrical Conductivity (%Δ)	Sources
CNTs	1–2	+35…50%/+20…30%	+40…70%	дo 10^6^ (percolation)	[27,31]
Graphene/graphene oxide	0.5–1	+25…40%/+15…25%	+50…120%	+10^3^…10^4^	[27]
Nanoclays (MMT, organo-MMT)	3–5	+15…25%/+10…15%	+20…30%	insignificant	[32]
Metal nanoparticles (Ag, Cu)	1–2	+10…20%/+5…10%	+30…50%	+10^2^…10^3^	[13]
Ceramic (Al_2_O_3_, SiO_2_, TiO_2_)	2–5	+20…35%/+15…20%	+60…90%	–	[24]
Hybrid (CNT + clay, graphene + oxides)	1–3	+40…60%/+25…35%	+100…150%	+10^3^…10^5^	[33,34]

Note. Values are given as typical ranges of relative changes (%Δ) based on the results of generalized literature data.

**Table 5:** The main methods of incorporating nanofillers into the polymer matrix.

Method	Process Essence	Advantages	Disadvantages and Limitations	Sources
Liquid technology	Dispersion of nanophases in a polymeric solution or monomer followed by drying	Homogeneous distribution; suitable for heat-sensitive polymers; microstructure control	Toxic solvents used; high energy consumption; slow solvent removal	[2,25]
Mechanical mixing in the melt	Intensive mixing of nanophases in a polymer melt followed by extrusion	High performance; industrial scalability; solvent-free	Possible aggregation of nanoparticles; limited dispersion quality without modification	[13,22,25]
In situ synthesis	Polymerization of monomer in the presence of nanofillers	Chemical bonding at the interface; high degree of intercalation; improved adhesion	Not suitable for all polymers; requires precise control of reaction conditions	[2,13,32]
Superficial functionalization	Chemical modification of nanophases with functional groups to improve compatibility	Improve adhesion and dispersion; reduce aggregation; control selective interaction	Additional synthesis steps; the need to select appropriate reagents and functionalization conditions	[2,13,25]

**Table 6:** Application of PNCs in various industries.

Area of Application	Examples of Nanofillers	Key Properties/Effects	Examples of Materials or Products	Sources
Construction	Nanoclay, oxide nanoparticles	Barrier, fire resistance, wear resistance	Coatings, thermal insulation, sealants	[11,24]
Aviation and automotive industry	CNTs, nanographene	Strength, weight reduction, electrical conductivity	Cladding, stabilizers, anti-static enclosures (Noryl GTX)	[14,24,28]
Medicine and biotechnology	Silver, zinc nanoparticles, fullerenes	Antibacterial, biocompatible, permeability control	Implants, membranes, delivery capsules	[11,33]
Electronics and sensors	CNTs, graphene, graphene oxide	Flexibility, electrical conductivity, transparency, sensitivity	Sensors, flexible electrical circuits, smart textiles	[14,24]

**Table 7:** Generalization of the properties of nanofillers for polymer composites.

Type of Nanofiller	Main Effects and Properties	Minimum Effective Concentration	Main Areas of Application	Potential Risks	Approximate Cost (€/kg)	Sources
Carbon (CNT, graphene, fullerenes)	Electrical conductivity, stiffness, barrier properties	0.1–1.0%	Electronics, aviation, sensors	Aggregation, toxicity	150–800	[12,14,24]
Metal (Ag, Cu, Au)	Antimicrobial, thermal conductivity, electrical conductivity	0.5–2.0%	Medicine, packaging, thermally conductive polymers	Price, ionic leaching	500–1500	[13,35]
Ceramic (SiO_2_, TiO_2_, ZnO, Al_2_O_3_)	UV resistance, chemical inertness, wear resistance	1.0–5.0%	Construction, protective coatings, optics	Agglomeration, low adhesion	20–120	[13,24]
Polymeric (dendrimers, polyaniline, gels)	Biocompatibility, flexibility, controlled adhesion	1.5–3.0%	Biomedicine, tissue engineering	Mechanical weakness, moisture	30–100	[11,35]
Hybrid (e.g., Ag-TiO_2_, graphene-Fe_3_O_4_)	Multifunctionality, synergistic effect	0.5–2.5%	Smart surfaces, biosensors, energy	Instability, complexity of synthesis	200–1000	[12,13,14]

**Table 8:** Technological and industrial challenges in the production of PNCs and possible ways to overcome them.

Challenge	Manifestations/Consequences	Possible Solutions	Sources
Nanoparticle aggregation	Agglomerate formation, reduced interfacial adhesion, structural defects	Surface functionalization, dispersants, ultrasonic or high-shear mixing	[2,29]
Scaling up the synthesis of nanofillers	High cost of CNTs, graphene, functionalized metal oxides; quality variability	Development of low-cost synthesis methods (hydrothermal, thermochemical, plasma technologies), industrial scaling	[2,13]
Processing complexity	Increase in viscosity, uneven distribution of nanophases in the matrix, need to adapt equipment	Optimization of extrusion/molding modes, application of solvent methods, in situ polymerization	[2,13]
Stability in time	Oxidation, aggregation or degradation of nanophases under humidity, UV or thermal stress	Protective coatings, nanoparticle encapsulation, surface modification with antioxidants	[13,29]
Economic barriers	High cost of the final product, limited implementation in mass production	Reducing the price of nanomaterials through scaling, combined hybrid nanofillers	[11,14]
Environmental and safety risks	Potential toxicity, lack of standards for safe use, difficulty in disposal	Toxicological studies, creation of a regulatory framework, development of biocompatible and biodegradable nanofillers	[11,13,14]

## Data Availability

No new data were created or analyzed in this study. Data sharing is not applicable to this article.

## Supplementary Materials

The following supporting information can be downloaded at: https://www.mdpi.com/article/10.3390/polym18172120/s1.
